# Rural Perspectives on Digital Health in Cardiovascular Care: Qualitative Study of Interviews With Rural and Rural-Serving Primary Care Providers and Cardiologists

**DOI:** 10.2196/77234

**Published:** 2025-11-07

**Authors:** Signe Burchim, Susan Miller, Kristin Beima-Sofie, Angela G Spencer, Brekken Selah, Elena Wadden, Adiya Jaffari, Monica Zigman Suchsland, Allison Cole, Steven Elrod, Margaret A Gehring, Ryan Gilles, Charles G Jose, Kelly McGrath, Russell T Baker, Chris T Longenecker

**Affiliations:** 1 Department of Global Health University of Washington Seattle, WA United States; 2 Department of Medicine University of Washington Seattle, WA United States; 3 Department of Family Medicine University of Washington Seattle, WA United States; 4 Providence Chehalis Family Medicine Chehalis, WA United States; 5 St. Mary's Health Cottonwood, ID United States; 6 Kootenai Clinic Coeur d'Alene Family Medicine Residency Coeur d'Alene, ID United States; 7 PeaceHealth Ketchikan, AK United States; 8 Clearwater Valley Health Orofino, ID United States; 9 School of Health and Medical Professions University of Idaho Moscow, ID United States; 10 Division of Cardiology Department of Medicine University of Washington Seattle, WA United States

**Keywords:** cardiovascular, CVD, digital health, heart disease, mHealth, remote patient monitoring, rural, telehealth

## Abstract

**Background:**

Digital health technologies, such as telehealth, remote patient monitoring, and smartphone apps, have the potential to reduce access disparities faced by rural patients with cardiovascular disease, but little is known about rural health care providers’ perspectives on adopting digital health in their practice.

**Objective:**

This study used diffusion of innovations theory as a guiding framework to interpret interview findings on rural and rural-serving health care providers’ perspectives on the use of digital health to deliver rural cardiovascular care.

**Methods:**

We conducted semistructured interviews with rural and rural-serving health care providers, including primary care advanced practice providers and physicians, as well as referring cardiologists from 6 primary care clinics in Alaska, Idaho, and Washington. We performed a directed content analysis of interview data informed by diffusion of innovations theory and identified emergent subthemes related to each of the 5 factors that influence adoption: relative advantage, compatibility, complexity, trialability, and observability.

**Results:**

Seventeen health care providers participated in this study. Participants described cycles of adopting and discontinuing the use of digital health in their practice. Participants identified advantages of digital health including reduced patient travel, the ability to leverage nonphysician health care workers, and the availability of objective patient data from remote patient monitoring. Compatibilities included increased patient adherence and follow-up and the ability to involve specialists in patient care. The trialability of digital health was described through experiences with remote patient monitoring and scaled-up use of telehealth during the COVID-19 pandemic, and participants observed the benefits of digital health in other disciplines and as patients. We also identified several disadvantages, incompatibilities, and complexities that may hinder the adoption of digital health technologies in rural practice, most of which were highlighted at the clinic and patient levels. These disadvantages, incompatibilities, and complexities included substandard equipment, inability to perform a physical examination, connectivity issues caused by poor internet and cell phone service, concerns about patient age and technical abilities, concerns about proper fit and distribution of remote patient monitoring equipment, and questions about billing and data management for digital health technologies.

**Conclusions:**

Rural health care providers recognize the many advantages of using digital health in caring for patients with cardiovascular disease but find that digital health is often complex and incompatible with their needs and the needs of their patients. There may be a disconnect between the potential of digital health and how it works in practice, as evidenced by the cycles of adoption and discontinuance of digital health technologies described by rural health care providers. Future rural digital health interventions in cardiovascular care should take into consideration specific complexities and incompatibilities in the rural context.

## Introduction

### Background

#### Rural Cardiovascular Health

Cardiovascular disease (CVD) is the leading cause of death in the rural United States and accounted for 1 in every 5 deaths in 2022 [1]. There are many ways to define rural in the United States, but rural areas are generally sparsely populated and located far from urban centers [2]. More than 2 decades of data show that rural Americans experience higher cardiovascular mortality rates than their urban counterparts [3,4]. A recent study found that the rural-urban disparity is worsening, with rural cardiovascular mortality rates 150% higher than urban rates in 2022 [5]. Furthermore, premature death rates from CVD were higher in rural areas than in urban areas from 2010 to 2022, highlighting serious rural-urban inequities [6].

Overall, rural populations have higher rates of cardiovascular risk factors, such as physical inactivity, tobacco use, diabetes, hypertension, and obesity [7-11]. Receiving proper care to manage cardiovascular risk factors and disease is critical to ensuring the health of rural populations, but rural-dwelling people face considerable social and structural barriers in accessing cardiovascular care [12]. More than 85% of rural counties do not have a cardiologist [13], resulting in rural patients relying on visiting cardiologists, local primary care providers, traveling long distances, and telehealth services to receive cardiovascular care [14]. One study found that more rural Medicare beneficiaries rely on generalist physicians and advanced practice providers compared with their urban counterparts, further underscoring the important role rural primary care providers have in the delivery of cardiovascular care [15]. In 2020, the American Heart Association released a call to action highlighting rural-urban disparities in cardiovascular health and cited exploring telehealth and other digital health technologies as potential solutions for improving rural cardiovascular health [12].

#### Digital Health Technologies in the Rural Setting

The use of digital health technologies, including mobile health (mHealth), health information technology, wearable devices, telehealth (or telemedicine), remote patient monitoring (or telemonitoring), and personalized medicine [16], may increase access to cardiovascular care for patients who live in rural areas. Advantages of using digital health to provide care include increased access to health information, improved communication with health care providers, personalized care, remote patient monitoring capabilities, and patient self-management [17]. Rural-dwelling people have less access to broadband internet than people living in urban areas [18], and while telehealth has become far more widespread due to the COVID-19 pandemic, uptake has been lower in rural than in urban areas [19-22]. A national study found that people in rural areas are less likely than their urban counterparts to use digital health technologies to communicate with their health care providers but are equally likely to own and use digital health technologies to manage their health [23].

Understanding the perspectives of rural health care providers is critical to understanding why rural digital health technology use lags behind urban use. Little is known about rural health care providers’ perspectives regarding the use of digital health technologies in the delivery of specialty care, including cardiovascular care [14,24].

#### Diffusion of Innovations Theory as a Guiding Framework

The Diffusion of Innovations (DOI) theory provides a useful framework for characterizing health care providers’ perspectives on digital health and whether those perspectives may support or hinder future adoption. Rogers [25] defined diffusion as “the process by which an innovation is communicated through certain channels over time among the members of a social system.” Potential adopters move through the diffusion-innovation process, the stages of which include (1) knowledge, or initial exposure to the innovation; (2) persuasion, or developing a positive or negative attitude toward the innovation; (3) decision, or engaging in activities that lead to choosing whether to adopt or reject the innovation; (4) implementation, or putting an innovation to use; and (5) confirmation, or seeking reinforcement about an innovation decision that has already been made. At each stage, the decision to adopt an innovation can be reversed later (a stage called discontinuance), or the decision to reject an innovation can be reversed. Five factors that influence whether an innovation will be adopted include relative advantage, compatibility, complexity, trialability, and observability. Relative advantage, compatibility, trialability, and observability are thought to be positively related to the rate of adoption, whereas complexity is negatively related to the rate of adoption. While other frameworks, such as the Technology Acceptance Model and the Unified Theory of Acceptance and Use of Technology, also offer a useful guide for understanding individual perspectives on digital health, we chose to use DOI for its emphasis on the role of the social system. A key component of DOI theory is the role of the opinion leader—an individual who influences others’ opinions about an innovation [25]. Opinion leaders’ reactions are critical to potential adopters, and social systems do not change without opinion leaders adopting innovations [26]. For the purpose of this study, we examined rural and rural-serving health care providers as opinion leaders in adopting the use of digital health technologies in the delivery of rural cardiovascular care.

### Objective

This study aimed to interpret health care providers’ perspectives on the use of digital health technologies to deliver rural cardiovascular care, using DOI theory as a guiding framework. We also identified potential facilitators and barriers to providers’ adoption of digital health to better inform future digital health interventions in rural cardiovascular care.

## Methods

### Study Design

This study was part of a formative research project [27] assessing barriers and facilitators to delivering rural cardiovascular care in the Mountain West and Pacific Northwest as part of the Global to Rural Innovation Network (GROW-Rural) project. A multidisciplinary team of public health researchers, social scientists, and clinicians developed semistructured interview guides. This study follows the COREQ (Consolidated Criteria for Reporting Qualitative Research) guidelines, where applicable [28].

### Participants and Recruitment

Participants were recruited through a partnership with the WWAMI Region Practice and Research Network (WPRN). The WPRN is a primary care, practice-based research network of clinics and clinical organizations in the 5-state WWAMI (Washington, Wyoming, Alaska, Montana, and Idaho) region. We invited all WPRN clinics located in rural or rural-serving counties to participate in this study. Rurality was determined using the 2023 Rural-Urban Continuum Codes (RUCCs). RUCCs 1-3 are classified as metropolitan (urban) counties, and RUCCs 4 and higher are classified as nonmetropolitan (rural) counties [29]. Clinics in metropolitan counties with an RUCC of 3 were considered rural-serving for the purposes of this study. We worked with WPRN practice champions, defined as providers who volunteer to facilitate research within participating WPRN member practices. WPRN practice champions identified and recruited colleagues to participate, and after completing this study, we asked participants to identify other colleagues they believed would be a good fit for the study. We sampled a range of health care providers who deliver cardiovascular services [30]. Our study population consisted of primary care providers, including physicians, nurse practitioners, and physician assistants, as well as referring cardiologists. Referring cardiologists were employed by the same health system as the rural clinics and traveled to provide itinerant care at those clinics. Some practice champions (SE, MAG, RG, CGJ, and KM) were also invited to serve as authors of the manuscript but were not involved in the data analysis process and did not have access to raw data.

### Data Collection

From December 2023 through October 2024, we administered surveys to collect demographic characteristics using REDCap (Research Electronic Data Capture; Vanderbilt University), a secure, web-based application hosted at the Institute for Translational Health Sciences at the University of Washington [31,32], and conducted semistructured interviews using Zoom, Microsoft Teams, and telephone. Interviews were pilot tested to refine the interview guide. The qualitative analysis team met after conducting 3 pilot interviews, and minimal adjustments were made; therefore, we chose to include data from pilot interviews in our analysis. We audio recorded and transcribed interviews with participant consent. Interviews lasted an average of 42 minutes and were conducted by 2 research scientists working for an urban public research university (SB and SM), both of whom had formal training and experience in qualitative methods and in working with rural populations. Interviewers completed a debrief report following each interview and noted first impressions and key information from the interview. The research team held meetings throughout the data collection process to discuss the data. We ceased data collection after determining that no new themes were discussed in interviews, thereby reaching saturation [33]. Topics of interview questions relevant to this analysis included how participants make use of technology in their practice with patients with CVD and thoughts on using a Bluetooth-enabled blood pressure cuff for remote patient monitoring. Survey questions on participant characteristics collected demographic data and asked about openness to using technology to manage CVD to help characterize our participants.

### Data Analysis

Transcripts were analyzed using a directed content analysis approach [34]. We first deductively coded segments of text using the 5 factors that influence adoption from Rogers’ [25] DOI theory, then inductively identified emergent themes within each factor. The first author coded all transcripts and discussed findings with other members of the research team. Qualitative coding and data management were performed using ATLAS.ti 24 (Scientific Software Development GmbH) and Microsoft Excel. We used descriptive statistics to analyze quantitative items in Microsoft Excel. The term digital health is used throughout this study to broadly refer to remote patient monitoring, the use of smartphones in health care, and telehealth.

### Ethical Considerations

The University of Washington Institutional Review Board determined this study (STUDY00018300) to be exempt from full review because it involved minimal risk to participants, with data collected through surveys and interviews. Each participant consented to participate in the study, with the understanding that they could withdraw at any time without consequences. All study data were deidentified and stored on a secure server. Participants were compensated with a US $50 Visa gift card for completing the survey and interview.

## Results

### Participant Characteristics

Seventeen health care providers from 6 different clinics were included in this analysis. Primary care providers accounted for three-fourths of our sample, with 10 primary care physicians (10/17, 58.9%) and 3 advanced practice providers (3/17, 17.6%). The remaining one-fourth were cardiologists (4/17, 23.5%). Our study sample consisted of more women than men (11/17, 64.7% women vs 6/17, 35.3% men), and participants’ average age was 47 years. All participants self-identified as White, and one participant self-identified their ethnicity as Hispanic/Latino (1/17, 5.9%). Most providers practiced in Idaho (12/17, 70.6%), followed by 3 in Alaska (3/17, 17.6%) and 2 in Washington (2/17, 11.8%). Participants’ clinics varied in rurality, with more than two-thirds (11/17, 64.7%) practicing at a clinic in a nonmetro county with an urban population of fewer than 20,000. Participants’ demographic characteristics are displayed in Table 1.

**Table 1:** Demographic characteristics of rural and rural-serving health care providers who manage cardiovascular disease (N=17).

Characteristic			Value
**Provider type, n (%)**			
	Cardiologist	4 (23.5)	
	Primary care physician	10 (58.9)	
	Advanced practice provider^a^	3 (17.6)	
**Gender, n (%)**			
	Man	6 (35.3)	
	Woman	11 (64.7)	
White race, n (%)			17 (100)
Hispanic/Latino ethnicity, n (%)			1 (5.9)
Age (years), mean (SD)			46.8 (11.4)
**State, n (%)**			
	Alaska	3 (17.6)	
	Idaho	12 (70.6)	
	Washington	2 (11.8)	
**Rural-Urban Continuum Code (RUCC), n (%)**			
	3–Metro: Counties in metro areas <250,000 population	4 (23.5)	
	4–Nonmetro: Urban population ≥20,000, adjacent to a metro area	2 (11.8)	
	7–Nonmetro: Urban population 5000-20,000, not adjacent to a metro area	3 (17.6)	
	8–Nonmetro: Urban population <5000, adjacent to a metro area	8 (47.1)	

^a^Advanced practice providers include nurse practitioners and physician assistants.

**Figure 1:**
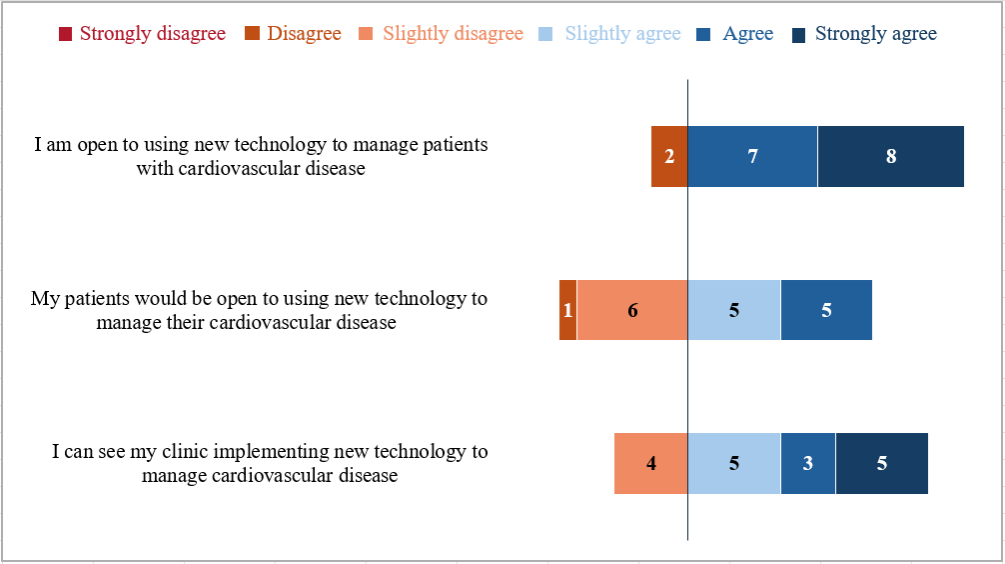
Rural and rural-serving health care providers’ perceptions of openness to using new technology in the management of cardiovascular disease (N=17).

Most participants agreed that they were open to using new technology to manage their patients with CVD (15/17, 88.2%). Participants were largely neutral about the openness of their patients and clinics to use new technology, with two-thirds slightly agreeing or slightly disagreeing (11/17, 64.7%) regarding their patients’ openness and half slightly agreeing or slightly disagreeing (9/17, 52.9%) regarding their clinics’ openness to implementing new technology. No participants strongly agreed that their patients would be open to using new technology (Figure 1).

### Five Factors Influencing Adoption

All participants had experience using digital health technologies in their practice, but their use was limited and inconsistent. Most participants discussed using some form of telehealth, while fewer discussed using remote patient monitoring technology or smartphones with their patients. In interviews, participants drew on past experiences with digital health and offered thoughts about adopting digital health in the future. Table 2 displays our operationalized definitions of the 5 factors for the purpose of this study. Participants described cycles of adoption and discontinuance of digital health technologies. Subthemes related to the factors that influence innovation adoption, both positively and negatively, are shown in Table 3 and are described in further detail in subsequent sections.

**Table 2:** Operationalized definitions of the 5 factors influencing adoption (from Diffusion of Innovations theory).

Factor	Operationalized definition
Relative advantage	The degree to which digital health technologies are better (or worse) than nondigital modalities in the delivery of rural care
Compatibility	The degree to which digital health technologies are consistent (or inconsistent) with the values, past experiences, and needs of rural care providers
Complexity	The degree to which digital health technologies are perceived as difficult to understand and use
Trialability	The degree to which digital health technologies may be experimented with on a limited basis
Observability	The degree to which the results of digital health technologies are visible to others

**Table 3:** Subthemes related to the 5 factors that influence adoption (from Diffusion of Innovations theory), derived from semistructured interviews with rural and rural-serving health care providers who manage cardiovascular disease.

Factor	Subthemes
Relative advantage	Decreased need for patient travel Ability to leverage nonphysician health care workers Availability of objective data from remote patient monitoring Digital health equipment is not up to standard, leads to unsatisfactory care
Compatibility	Increased patient compliance and follow-up Direct involvement from cardiologists or emergency providers in patient care Concerns about patient age, ability, and access Inability to perform physical examinations
Complexity	Connectivity issues Concerns about fit and distribution of remote monitoring equipment Questions about billing and data management
Trialability	Remote blood pressure monitoring programs Use of other remote patient monitoring technology Telehealth during the COVID-19 pandemic
Observability	Observation of telehealth in cardiology and other disciplines Use of telehealth as a patient

### Theme 1: Relative Advantage

For this analysis, we defined relative advantage as the degree to which digital health technologies are better than nondigital modalities in the delivery of rural cardiovascular care. The identified subthemes address issues that seemed to be essential or most likely to represent direct advantages or disadvantages of using digital health technology. We found 3 subthemes related to the relative advantage of digital health in caring for patients with CVD, including the decreased need for travel, increased use of nonphysician health care workers, and availability of objective data for patient management. We also identified one relative disadvantage to using digital health: digital health is not up to standard and can lead to substandard care.

#### Subtheme 1a: Decreased Need for Patient Travel

Participants felt digital health was useful for decreasing the need for patients to travel long distances for care that can be managed remotely and specifically cited the desire for a telehealth heart failure clinic. Travel and weather were concerns for participants, who felt digital health may help alleviate transportation challenges for both patients traveling to see providers and providers traveling to see patients. One participant described the difficulties of traveling to and from remote locations in Alaska during the winter:

I'd love to be able to do more of this [digital health] for people on [a remote] island because we can't get there all year long because the weather is too bad. That's another barrier. You can only go certain months because it gets just too treacherous to travel by air over there. And they don't want to travel by boat either because the water is also rough. So part of the year they just are out of care.

#### Subtheme 1b: Ability to Leverage Nonphysician Health Care Workers

Participants appreciated that digital health provided opportunities to involve nonphysician health care workers in the delivery of telehealth and remote patient monitoring, as this can help ease physicians’ busy schedules and reach more patients. Some clinics already had programs in place, such as one clinic that used community health workers for a remote blood pressure monitoring program to “address the disparities” and reach people “outside the bricks and mortar of our buildings.”

#### Subtheme 1c: Availability of Objective Data From Remote Patient Monitoring

Participants also appreciated that remote patient monitoring provided objective data from outside the clinic setting and specifically noted that remote blood pressure monitoring can help clinicians accurately titrate medications, as blood pressure readings are often different at home compared with clinic readings. Participants who had not used remote patient monitoring recognized its benefits and spoke favorably about using it in the future.

[Remote blood pressure monitoring] would be really helpful […] I think it, obviously, would give us a much better picture of the patient's condition if we had a reliable source of what their blood pressure is running outside of that one moment in time, just because blood pressure fluctuates so much depending on what people are doing, and stress levels, and whether or not they're taking their medicines.

#### Subtheme 1d: Digital Health Equipment Is Not Up to Standard, Leads to Unsatisfactory Care

Speaking specifically about experiences with telehealth, participants felt that a disadvantage of using digital health was that it can lead to unsatisfactory patient care due to issues with technology not working correctly. Participants also noted the digital health equipment was not always up to their standards and, in some cases, had regressed in quality compared with several years ago. One cardiologist shared that telehealth visits were often a hassle to conduct:

Trying to coordinate telehealth sometimes is more work than it's worth. It's a big uphill battle. Despite lining everything up, it doesn't have the same flow as your office, so a 15 or 20-minute visit oftentimes takes 45 or 50 minutes, so it's very challenging. It disrupts your patients here. It sometimes doesn't feel like it's worth the hassle. I mean, as much as you empathize for the patients and you want to provide a convenience for them, I think that our practice just is not as streamlined as we wish it was.

### Theme 2: Compatibility

We defined compatibility as the degree to which digital health technologies are consistent with the values, past experiences, and needs of rural health care providers. The identified subthemes address issues that seemed valuable to participants but were distinct from the essential advantages likely to result from their use. We identified 2 subthemes highlighting the compatibility of using digital health in the delivery of rural cardiovascular care: increased patient compliance and follow-up, and the ability to involve specialist physicians and emergency providers in patients’ care.

Additionally, we identified 2 subthemes related to the incompatibility of using digital health to deliver rural cardiovascular care: provider concerns about patient age and ability to use digital health technologies, and the inability to perform physical examinations.

#### Subtheme 2a: Increased Patient Compliance and Follow-Up

Participants with experience using telehealth felt that digital health technology increased patient compliance and reduced the likelihood of losing a patient to follow-up. They regularly used telehealth as a tool for following up with patients and keeping them on track with their care plan. One participant shared their perspective on the benefits of telehealth:

In chronic disease management and follow-up, telehealth visits can be very beneficial for both me checking on my patients where they’re at, catching a heart failure exacerbation before it’s hospital admission time, when a patient can’t come or would normally cancel an appointment, and we lose the follow-up.

#### Subtheme 2b: Direct Involvement From Cardiologists or Emergency Providers in Patient Care

Participants appreciated that digital health technologies provide opportunities for involvement, such as through telehealth visits, by providers who are more experienced in emergency or complex cardiovascular cases, including heart failure transplant–boarded cardiologists, electrophysiologists, and emergency providers.

So if I am sitting in [rural] Idaho in the little ED [emergency department] and I have an acute cardiac patient, we have the ability to wheel in an iPad and have a telehealth visit, either with the ED provider or the cardiologist on call to talk to the patient, really look at the patient, how sick they are and provide recs right then and there. And so that acute care is amazing.

#### Subtheme 2c: Concerns About Patient Age, Ability, and Access

Participants’ concerns about their patients’ older age and general ability to use technology highlight how digital health can be incompatible with the delivery of rural cardiovascular care. Some participants also shared that not all of their patients have the ability to access or use digital health tools:

The most recent generations of CGMs [continuous glucose monitors] require smartphone integration, whereas older ones have their own little magic wand you hold up to them. And there are some of our patients who need the older system, they’re just like, ‘I cannot use this cell phone integration thing.’ Is that the majority of patients? No. But sometimes they just don’t have that cell phone or they don’t have access to a plan.

#### Subtheme 2d: Inability to Perform Physical Examinations

The inability to perform a physical examination using digital health was incompatible with participants’ needs in providing patient care. One participant shared that she “hates” telehealth because she likes “laying my hands on a patient and basically evaluating them, listening to their heart, talking to them, checking their blood pressure, that kind of stuff.” She later noted that she likes using telehealth for ongoing patient management, such as ensuring correct dosing for blood pressure medications.

### Theme 3: Complexity

We defined complexity as the degree to which digital health technologies are perceived as difficult to understand and use. Subthemes related to the complexity of digital health technologies highlighted the difficulties and concerns participants have experienced or felt were likely to result from using digital health in their practice. They were largely concerned with difficulties at the patient and clinic levels. We identified 3 subthemes related to the complexity of digital health: connectivity issues, concerns about the fit and distribution of remote monitoring equipment, and questions about billing and data management.

#### Subtheme 3a: Connectivity Issues

Participants shared past experiences with connectivity issues related to unreliable internet and telephone access, both in the clinic and for their patients at home. They described instances in which issues with internet and technology led to substandard visits and sometimes interrupted the flow of their schedules with in-clinic patients. One cardiologist felt that fewer than half of their telehealth visits ran smoothly:

I mean, it’s probably less than 50% goes smoothly as far as a patient logs on, you have a visit, and it accomplishes the goal. Probably at least half the time when we weren’t bringing them into the office, the internet wouldn’t work. The video was such poor quality. It would disconnect and then you were calling on the phone and the next. It just took a lot more time. Ultimately at the end of the day, you didn’t accomplish what you were really hoping for. As a provider, I felt like that was a substandard visit.

#### Subtheme 3b: Concerns About Fit and Distribution of Remote Monitoring Equipment

Participants were concerned about the distribution of remote patient monitoring equipment and ensuring that the equipment they provided to patients fits correctly, is used correctly, and is returned by patients. One primary care physician shared that her clinic had to stop offering remote treatment of bilirubin because patients were not returning the equipment. She felt that remote patient monitoring would work best if patients were able to keep the equipment:

We do give out blood pressure cuffs, but we basically have had a difficult time I think over time having people return equipment. […] But if it was something that was given to the patient, I think that would be great.

#### Subtheme 3c: Questions About Billing and Data Management

The complexity of managing remote patient monitoring data and difficulties with billing for digital health were clinic-level concerns for participants. They were worried about the volume of remote patient monitoring data, taking responsibility for it, and entering it into the electronic health record (EHR). Participants mentioned that billing for digital health is more restrictive than for in-person visits. They also noted that restrictions on billing and providing telehealth across state lines were less restrictive during the COVID-19 pandemic but have since returned to stricter regulations. One primary care physician noted that the modern practice of medicine requires one to consider billing for remote patient monitoring:

I think probably the biggest barrier is how does that information get into the EHR. And then, when are you monitoring that? Who’s monitoring it? If somebody’s blood pressure is 240, when does that get seen and what is the advice, having time set aside to do that, is there a way to bill for it that? Which I hate to think about that, but that’s kind of the modern practice of medicine and employed practice.

### Theme 4: Trialability

We defined trialability as the degree to which digital health technologies may be experimented with on a limited basis. Subthemes related to trialability highlighted participants’ past or current experiences using digital health tools. We identified 3 subthemes related to the trialability of digital health: use of remote blood pressure monitoring programs, use of other remote patient monitoring technology (ie, continuous glucose monitors [CGMs], Zio patches, and Holter monitors), and use of telehealth during the COVID-19 pandemic.

#### Subtheme 4a: Remote Blood Pressure Monitoring Programs

As noted above in the relative advantage section, participants who had used remote patient monitoring in the past generally reported positive experiences. One participant described their experience using remote blood pressure monitoring to manage medication:

I’ve had a couple of patients who have pretty normal looking blood pressures when they’re in office, but then I see that they’re markedly hypertensive most of the time when they’re at home. And so I’ve actually increased meds for people more often than I’ve decreased, I think.

#### Subtheme 4b: Use of Other Remote Patient Monitoring Technology

Participants had used several other types of remote monitoring technology in their practice, including Zio patches, CGMs, and Holter monitors. The success of using these technologies was mixed. As noted above in subtheme 2c, some participants felt their patients had difficulties using smartphone-integrated remote patient monitoring technology. Other participants, however, had success using different remote patient monitoring technologies with their patients. One primary care physician described the benefits of being able to monitor data from patients’ CGMs and felt those benefits would translate to using remote blood pressure monitoring:

[…] Continuous glucose monitors for patients change everything with diabetes. I mean, it’s so much easier when I can just log on and see exactly what is going on. I think ambulatory blood pressure monitoring would be amazing.

#### Subtheme 4c: Telehealth During the COVID-19 Pandemic

Participants who used telehealth during the COVID-19 pandemic had mixed feelings about its success. Many participants noted that telehealth use was higher during the pandemic and has since declined. One advanced practice provider noted, “they aren’t doing a lot of telehealth anymore for cardiac patients, so there is that delay” in delivering cardiovascular care. Some participants were very satisfied with their experiences using telehealth and wished they could conduct telehealth visits as frequently as they did during the pandemic. Others noted frequent technical difficulties (highlighted above in the incompatibility and complexity sections). One cardiologist shared that he and some of his colleagues abandoned telehealth during the pandemic due to difficulties:

During COVID, I found that trying to do telemedicine with patients in their home was not worth it. I abandoned it, most of us did.

### Theme 5: Observability

We defined observability as the degree to which the results of digital health technologies are visible to others. Subthemes related to observability highlighted opportunities for participants to see successful digital health programs. We identified 2 subthemes related to observability: observation of telehealth in cardiology and other disciplines, and use of telehealth as a patient.

#### Subtheme 5a: Observation of Telehealth in Cardiology and Other Disciplines

Participants who had seen heart failure telehealth clinics through cardiology groups spoke highly of such programs, and some expressed a desire to have these programs in their community to reach more patients. Participants had also seen telehealth used to deliver behavioral health care. One participant shared that primary care providers in his clinic observed the benefits of having psychiatry support for complex cases and would like more telehealth access to specialty care:

[…] I think all of our patients and our providers are starving for access to specialty care. So we actually implemented adult and pediatric psychiatry in 2009 […] which has had a huge impact on the population. At least the high needs population, like the severely mentally ill patients that were being managed by primary care like myself. So the primary care team really appreciates things on those harder problems.

#### Subtheme 5b: Use of Telehealth as a Patient

In addition to observing successful telehealth in cardiology and other disciplines, participants also observed successful telehealth programs as patients. One participant, who was not happy with his experience using telehealth in his own practice, mentioned using telehealth successfully as a patient and noted that the technology he used as a patient worked very well, but he does not feel that he has provided that level of care to his rural patients.

Although that being said, as a patient, I've done telehealth through [urban healthcare facility], and my experiences were great. I don't know if it's because I was in a place and understood how to use technology, but they were able to share screens, and I thought it was very effective. […] I don't feel like I've provided the same service to our rural areas.

## Discussion

### Overview

This study used DOI theory as a guiding framework to interpret the perspectives of rural and rural-serving primary care providers and cardiologists toward using digital health in the delivery of cardiovascular care. Through this framework, we found that (1) participants identified several barriers and facilitators to using digital health in rural cardiovascular care, and (2) there may be a disconnect between the potential of digital health and how it works in practice, as evidenced by cycles of adoption and discontinuance of digital health technologies. Most participants indicated they were personally open to using digital health in their practice and identified many advantages to using digital health. They sometimes felt it was compatible with their needs and values, had opportunities to trial digital health technologies, and observed successful digital health in other practices. These results are consistent with findings from the broader DOI literature, which shows that compatibility, trialability, and observability of innovations are positively related to the rate of adoption [25]. Participants felt that their patients and clinics would be less open to adopting digital health. They also identified ways in which digital health had disadvantages and was complex and incompatible with their needs and values and those of their patients, all of which are thought to be negatively related to the rate of adoption per DOI literature [25].

Our findings suggest that digital health has the potential to increase access to cardiovascular care for rural patients and assist health care providers in delivering higher-quality care to their patients through this increased access. Findings indicating providers’ openness to digital health were consistent with the many advantages and compatibilities discussed in interviews, including the decreased need for travel, the ability to leverage nonphysician health care workers, the availability of objective data from remote patient monitoring, increased patient compliance and follow-up, and increased access to specialized health care providers.

Notably, our findings highlighted a disconnect between participant openness and consistent adoption of new digital health technology. Participants often found themselves in the confirmation stage of the diffusion-innovation process—seeking reinforcement for adoption decisions already made—and described instances of discontinuance, in which they referred to digital health programs in the past tense or spoke directly about abandoning them. Oftentimes, participants who had abandoned the use of digital health in the past adopted it again at a later date.

While we examined health care providers as opinion leaders in this study, we also note that participants’ perspectives on digital health were informed by their experiences with their patients and the clinics where they work. Participants indicated that they were less convinced of their patients’ and clinics’ openness to using new technology in the management of CVD than they were of their own. This is consistent with interview findings in which participants described disadvantages, complexities, and incompatibilities at the patient and clinic levels. Notable patient- and clinic-level incompatibilities and complexities included concerns about patients’ ability to effectively use digital health technologies, the quality of the available equipment, and digital health sometimes leading to substandard care.

This disconnect between the acknowledged advantages of providing digital cardiovascular care and how it works in rural practice is consistent with past studies. A scoping review examining the impact of digital health on bridging the rural health care gap found that, although digital health tools have the potential to increase access and improve outcomes for rural communities, there are critical barriers that must be addressed [35]. One study found that rural health systems leaders noted that telehealth has not been used to its potential in the delivery of specialty care, including cardiovascular care [14]. A study of rural primary care providers in the Midwest found that telehealth may not align with the needs of rural communities and should be optimized to address limitations of rural practice. In this study, only one-third of providers agreed that telehealth connects patients to better specialty care, whereas 90% agreed that it has the potential to make that connection [24]. Other studies showed that rural health care providers are interested in using mHealth technology with their patients but have limited time to learn about emerging technologies and voiced concerns about their patients’ ability to use mHealth technology [36,37].

While, in theory, digital health can increase access to cardiovascular care for rural patients, the potential to exacerbate existing health inequities must be acknowledged [38]. This study highlights the need to design rural digital health interventions with the needs of rural patients in mind. Patient-level considerations should include how digital health interventions can be designed to accommodate varying levels of digital health literacy, limited internet and smartphone access, and the need for patient education. Furthermore, future rural digital health interventions should consider the needs of rural health care providers and the limitations of their clinics. Such interventions should take care not to increase the workload of rural health care providers or compromise their ability to provide quality patient care. In addressing digital health equity, one should also consider the potential role of implicit bias in providers’ perceptions of patient barriers to using digital health. In designing a framework for digital health equity, Richardson et al [39] proposed the concept of implicit tech bias, which they describe as “the impact that unconscious perceptions of an individual’s digital literacy, technology access, and attitudes toward use have on clinician willingness to enroll and engage individuals with digital health care tools.” Health care providers and researchers should be careful to not predetermine whether a rural patient would be a good candidate for a digital health intervention.

### Strengths and Limitations

A strength of this study is its qualitative design, which allowed for an in-depth analysis of health care providers’ perspectives on the barriers and facilitators to using digital health in their practice. A second strength is that the inclusion of primary care physicians, advanced practice providers, and cardiologists allowed us to obtain the perspectives of health care providers who deliver various levels of cardiovascular care. This study is not without limitations. The DOI theory lends itself to a pro-innovation bias, and for the purpose of this study, it suggests that all rural health care providers should consider adopting digital health technologies to deliver cardiovascular care to their patients. Through this analysis, it became clear that digital health is not always the best method for delivering cardiovascular care, and health care providers should consider the needs of each individual patient. This study examines only the opinions of health care providers and does not account for the perspectives of rural patients. We also recognize that while there are many digital health technologies available, our analysis focused only on remote patient monitoring, the use of smartphones in health care, and telehealth. Although the qualitative methods used in this study allowed for an in-depth analysis of participants’ points of view, the data are not widely generalizable and do not represent the vast spectrum of rural communities. Furthermore, our sample was not racially or ethnically diverse, partially due to the demographics of the counties where our participants practiced, which have majority non-Hispanic White populations.

### Conclusion

Worsening rural cardiovascular health outcomes and a decreasing supply of rural cardiologists highlight the need for creative solutions to provide rural cardiovascular care, including digital health technologies. Rural health care providers recognize the many advantages of using digital health in caring for their patients with CVD but find that digital health is often complex and incompatible with their needs and the needs of their patients. There may be a disconnect between the potential of digital health and how it works in practice, as evidenced by the cycles of adoption and discontinuance described by rural health care providers. Future rural digital health interventions in cardiovascular care should take into consideration specific complexities and incompatibilities in the rural context.

## Data Availability

The datasets generated or analyzed during this study are not publicly available due to participant confidentiality but are available from the corresponding author on reasonable request.

## Appendix

Multimedia Appendix 1 Semistructured interview guide and technology survey questions.

## Abbreviations

CGM: continuous glucose monitors
COREQ: Consolidated Criteria for Reporting Qualitative Studies
CVD: cardiovascular disease
DOI: diffusion of innovations
EHR: electronic health record
GROW-Rural: Global to Rural Innovation Network
mHealth: mobile health
REDCap: Research Electronic Data Capture
RUCC: Rural-Urban Continuum Code
WPRN: WWAMI Region Practice and Research Network
WWAMI: Washington, Wyoming, Alaska, Montana, and Idaho
