# Supplementary material for: Rural Perspectives on Digital Health in Cardiovascular Care: Qualitative Study of Interviews With Rural and Rural-Serving Primary Care Providers and Cardiologists
Source: J Med Internet Res. 2025 Nov 7;27:e77234. doi: 10.2196/77234 (PMC12639339; doi:10.2196/77234)
Supplement: Multimedia Appendix 1 [file jmir_v27i1e77234_app1.pdf]

GROW-Rural Aim 1 Technology Questions and Semi-structured Interview Guide

Technology survey questions (Rated from 1 – 6, strongly disagree to strongly agree):

1. I would be open to using new technology (i.e., a phone application) to manage my patients with cardiovascular disease.
   a. Strongly disagree
   b. Disagree
   c. Slightly disagree
   d. Slightly agree
   e. Agree
   f. Strongly agree
2. My patients would be open to using new technology (i.e., a phone application) to manage their cardiovascular disease.
   a. Strongly disagree
   b. Disagree
   c. Slightly disagree
   d. Slightly agree
   e. Agree
   f. Strongly agree
3. I can see my clinic implementing new technology to manage cardiovascular disease.
   a. Strongly disagree
   b. Disagree
   c. Slightly disagree
   d. Slightly agree
   e. Agree
   f. Strongly agree

Semi-structured interview questions

(**For practice champions, medical directors and other clinical leaders):** How does your organization (or clinic) use technology when caring for your rural patients with cardiovascular disease?

(For clinicians): How do you use technology when caring for your rural patients with cardiovascular disease? (e.g., provider-to-patient, provider-to-provider, remote monitoring, mHealth applications, etc.)

a. What would you need to make better use of technology with your patients?

(For all): As you know, innovations in rural cardiovascular care are being developed around the world. For example, we are testing a heart failure intervention in Brazil that we think might be useful in the rural Northwest. In this intervention, patients with high blood pressure are given tools to monitor it – a Bluetooth-enabled blood pressure cuff for home use and a smartphone app that would share the readings with their healthcare team.

Would you be interested in implementing this innovation from Brazil, or another from a low- or middle-income country?

1. **(If there's time):** How would you try to integrate this new program into your workflow?
    a. What barriers do you think you would run into?
    b. What assistance would you need to implement it?
2. How did telehealth change in your office during and after the pandemic, if it did?
3. Did telehealth options make you feel less burnout? (probe: How so?)

(If there's time): I also have a few more questions about an intervention our team plans to implement. As I mentioned, one of our research aims is to adapt a digital toolkit for heart failure that uses a smartphone app along with tools to monitor patients' blood pressure and weight remotely (a scale and blood pressure cuff that are internet-enabled).

1. Would you tell me about your experience with the use of smartphone apps and mobile health interventions to help patients?

2. What do you think about patients' use of smartphone apps or technology for remote monitoring as part of treatment?

3. Do you use any of these technologies with patients currently? Which?

4. Given that you only know a little about the intervention at this point, do you think you might use it with patients?
    Probes:
    a. Why or why not?
    b. Do you personally feel capable?
    c. Given what you know at this point, do you think your clinic could handle this type of intervention?

5. What would help providers make the most use of the patient-facing technologies I mentioned? (probe for how providers would like to be informed about data collected by devices and how they would want technologies to interface with the medical record)
